# Supplementary material for: Inhibitory Effects of Breast Milk-Derived Lactobacillus rhamnosus Probio-M9 on Colitis-Associated Carcinogenesis by Restoration of the Gut Microbiota in a Mouse Model
Source: Nutrients. 2021 Mar 30;13(4):1143. doi: 10.3390/nu13041143 (PMC8065529; doi:10.3390/nu13041143)
Supplement: Supplementary file 1 [file nutrients-13-01143-s001.pdf]

		tumor number	averaged size (mm2)	size (mm2)						
1	Vehicle	0								
2	Vehicle	0								
3	Vehicle	0								
4	Vehicle	0								
5	Vehicle	0								
6	Vehicle	0								
7	Vehicle	0								
8	Vehicle	0								
9	Vehicle	0								
10	Vehicle	0								
1	AOM+DSS	3	2.51	2.30	1.77	3.46				
2	AOM+DSS	4	3.18	3.76	2.52	3.59	2.83			
3	AOM+DSS	6	2.77	2.50	2.54	2.49	4.15	2.06	2.88	
4	AOM+DSS	5	3.90	3.90	5.33	2.90	3.49	3.90		
5	AOM+DSS	4	3.99	2.66	4.52	5.90	2.88			
6	AOM+DSS	2	2.48	2.70	2.27					
7	AOM+DSS	7	3.80	4.90	2.54	4.60	4.15	5.72	2.70	2.01
8	AOM+DSS	2	4.00	3.80	4.20					
9	AOM+DSS	5	2.50	1.77	3.14	3.20	2.11	2.27		
10	AOM+DSS	3	3.93	3.21	3.87	4.70				
1	AOM+DSS+M9	0								
2	AOM+DSS+M9	1	1.49	1.49						
3	AOM+DSS+M9	0								
4	AOM+DSS+M9	1	1.21	1.21						
5	AOM+DSS+M9	2	2.50	2.34	2.65					
6	AOM+DSS+M9	0								
7	AOM+DSS+M9	1	1.80	1.80						
8	AOM+DSS+M9	0								
9	AOM+DSS+M9	0								
10	AOM+DSS+M9	0								

Supplementary table S1: The number and the size of polyps are listed in the table.

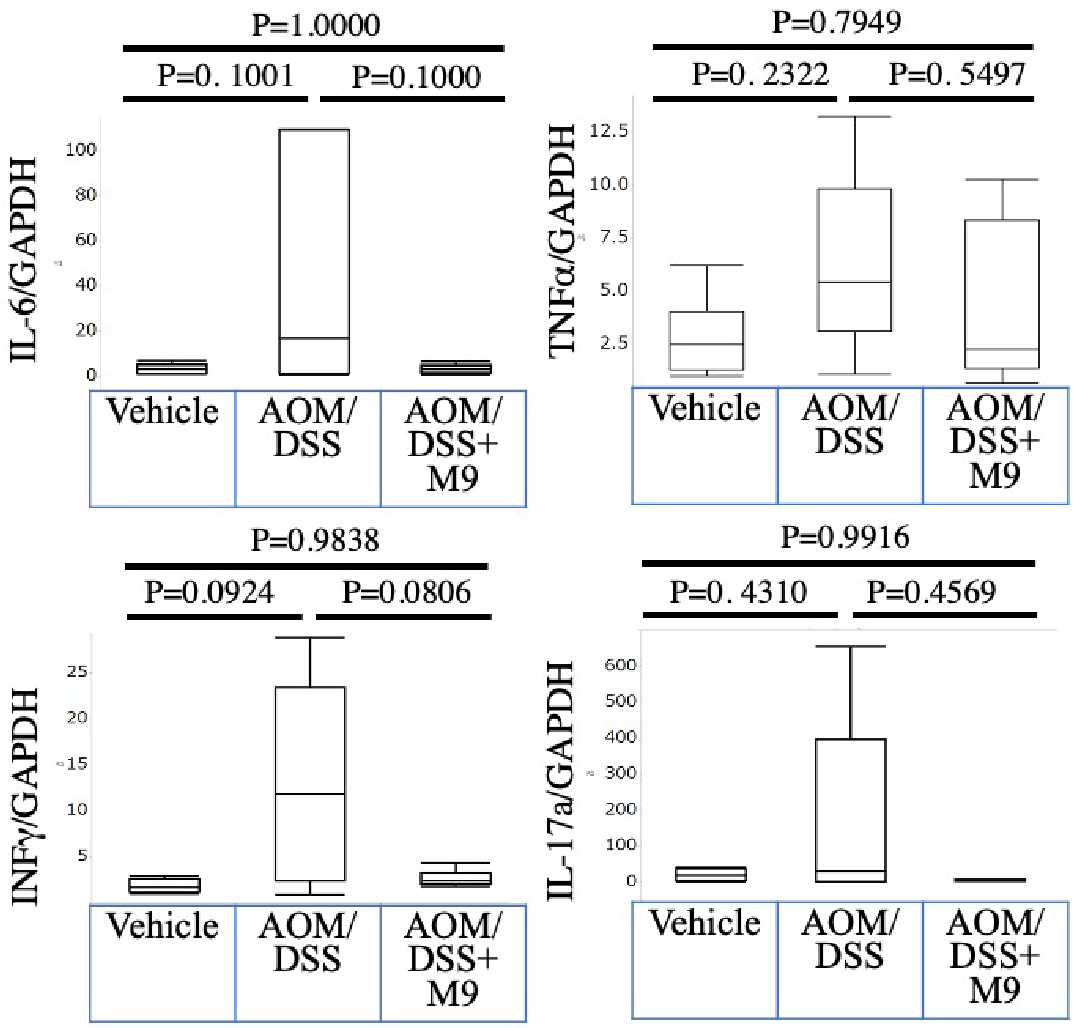

Supplementary Figure S1: Realtime RT-PCR of the cytokines IL-6, IL-17a, TNF-α, and INF-γ in distal colon. (n=6) Mouse GAPDH was used as endogenous control.

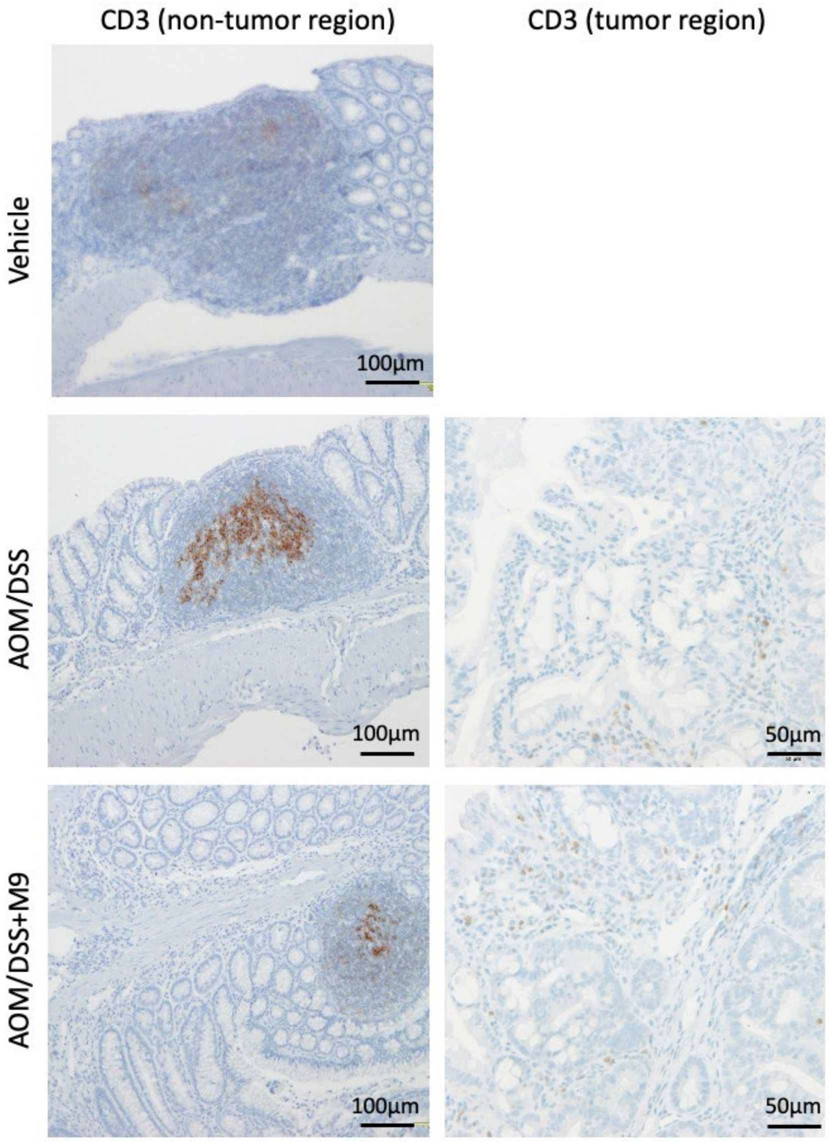

Supplementary Figure S2. Representative image of immunohistochemistry staining of CD3 in non-tumor and tumor area. The scale bar indicates 100 μ m and 50 μ m, respectively.
